# A quantitative method for determining spatial discriminative capacity

**DOI:** 10.1186/1475-925X-7-12

**Published:** 2008-03-10

**Authors:** Zheng Zhang, Vinay Tannan, Jameson K Holden, Robert G Dennis, Mark Tommerdahl

**Affiliations:** 1 Department of Biomedical Engineering, University of North Carolina School of Medicine, Chapel Hill, NC, USA

## Abstract

**Background:**

The traditional two-point discrimination (TPD) test, a widely used tactile spatial acuity measure, has been criticized as being imprecise because it is based on subjective criteria and involves a number of non-spatial cues. The results of a recent study showed that as two stimuli were delivered simultaneously, vibrotactile amplitude discrimination became worse when the two stimuli were positioned relatively close together and was significantly degraded when the probes were within a subject's two-point limen. The impairment of amplitude discrimination with decreasing inter-probe distance suggested that the metric of amplitude discrimination could possibly provide a means of objective and quantitative measurement of spatial discrimination capacity.

**Methods:**

A two alternative forced-choice (2AFC) tracking procedure was used to assess a subject's ability to discriminate the amplitude difference between two stimuli positioned at near-adjacent skin sites. Two 25 Hz flutter stimuli, identical except for a constant difference in amplitude, were delivered simultaneously to the hand dorsum. The stimuli were initially spaced 30 mm apart, and the inter-stimulus distance was modified on a trial-by-trial basis based on the subject's performance of discriminating the stimulus with higher intensity. The experiment was repeated via sequential, rather than simultaneous, delivery of the same vibrotactile stimuli.

**Results:**

Results obtained from this study showed that the performance of the amplitude discrimination task was significantly degraded when the stimuli were delivered simultaneously and were near a subject's two-point limen. In contrast, subjects were able to correctly discriminate between the amplitudes of the two stimuli when they were sequentially delivered at all inter-probe distances (including those within the two-point limen), and improved when an adapting stimulus was delivered prior to simultaneously delivered stimuli.

**Conclusion:**

Subjects' capacity to discriminate the amplitude difference between two vibrotactile stimulations was degraded as the inter-stimulus distance approached the limit of their two-point spatial discriminative capacity. This degradation of spatial discriminative capacity lessened when an adapting stimulus was used. Performance of the task, as well as improvement on the task with adaptation, would most likely be impaired if the cortical information processing capacity of a subject or subject population were systemically altered, and thus, the methods described could be effective measures for use in clinical or clinical research applications.

## Background

The capacity of a human subject to spatially resolve tactile stimuli delivered to the skin has traditionally been investigated by measuring the smallest distance between two tactile stimuli at which they evoke two distinct percepts [1]. Typically, the two-point discrimination (TPD) test has been widely used in clinical diagnoses as well as scientific studies. Along with its popular applications, however, TPD has been criticized as being imprecise for several reasons. First, it has been discussed that as the distance between two points varied, the perceptual patterns may gradually change. Tawney [2] stated that there were some intermediate sensations between the perception of one point and that of two points. As a result, the "first perception" of two points measured as TPD might provide an inaccurate measure of the minimum space of tactile spatial resolution whereas the "middle sensations" may represent the actual consciousness of spatiality [2,3]. Second, since different subjects adopted distinct criteria for defining two points, the responses were based to a great extent on the subject's experience. As a result, a large variability between subjects has been observed. Craig and Johnson [4] quoted a study in which Valentin and collaborators found that the TPD measures were highly inconsistent across all subjects, with nearly a four-fold difference in thresholds observed on the same region of the body. Third, traditional TPD tests involve a number of non-spatial cues which confounded subject discrimination. For instance, Tichener [5] found that in the objective TPD tests which employed one-point as well as two-point stimulation, subjects felt that the perceived intensity of one point was always stronger than that of two points. The above-described arguments suggest that the subjective TPD threshold might not provide a consistent and reliable measure of tactile spatial resolution. For these reasons, we sought to develop a more objective measure of spatial discrimination capacity.

Alternative methods have been developed to substitute for the traditional TPD test. Tannan et al. [6-8] presented a novel Two-Point Stimulator (TPS) which was capable of delivering two identical vibrotactile stimuli simultaneously at two discrete skin sites with variable distances on a trial-by-trial basis. By way of automated stimulus control and delivery, the TPS enabled a faster and more accurate administration of two-point measurement than previous TPD devices. However, in these particular studies, the discrimination test was still based on personal subjective criteria. Similarly, a number of other studies have demonstrated that grating orientation discrimination is a well-established and reproducible measurement of tactile spatial acuity on the finger pad [9-11]. However, it was argued that there might be substantial anisotropy on the finger pad which was related to spatial sensitivity and might permit subjects to discriminate grating orientation on the basis of intensive rather than spatial cues [10]. Additionally, a subject's orientation discrimination capability is typically assessed by interpolating the groove width with 75% correct responses [12,13]. Thus, in order to have enough values for interpolation, the percentages of accurate responses of several gratings with different groove widths need to be measured for each subject.

Recently, Tannan et al. [14] measured subjects' amplitude discrimination between two simultaneous 25 Hz vibratory stimuli delivered to the dorsum surface of the hand. The result indicated that amplitude discrimination became worse when the two stimuli were positioned relatively close together and was significantly degraded when the probes were within a subject's two-point limen. This impairment of amplitude discriminative capacity with decreasing inter-probe distance led the authors to hypothesize that the metric of amplitude discrimination could provide a means of objective and quantitative measurement of spatial discrimination between two-point on the skin. Such a measure could be used for objective evaluations of subject populations whose cortical information processing capacity is systemically altered or different from healthy control populations. In addition to assessing simple spatial discriminative capacity, slight modifications of stimulus conditions could reveal other aspects of a subject's central nervous system, based on predicted cortical-cortical interactions that result from these different stimulus conditions.

To investigate the above-described hypothesis, a modified Bekesy protocol was used to assess a subject's ability to discriminate a constant amplitude difference between two 25 Hz flutter stimuli as the stimuli were tracked to more proximal skin sites on the hand dorsum. Although comparable to an amplitude discrimination task which measures the minimum discriminable amplitude difference between two simultaneously delivered stimuli [14], the current protocol was unique in that the amplitude difference was constant and well above the average threshold amplitude difference limen (reported in previous studies [14,15]), and the inter-stimulus distance was modified on a trial-by-trial basis based on the subject's performance. The inter-stimulus distance metric obtained from the study appears to be fairly robust across the subjects studied thus far (i.e., low variance between individual performance) and can be obtained relatively quickly (about three minutes).

## Methods

Ten subjects participated in this experiment. They were naïve both to the study design and issue under investigation. All experimental procedures were reviewed and approved in advance by an institutional review board.

The tactile stimuli used in this study were sinusoidal vertical skin displacements delivered by a novel dual-site vibrotactile stimulator (details about the CM-1 stimulator are described in a recent report; [14]). The CM-1 dual-site stimulator is capable of delivering two tactile stimuli simultaneously or sequentially at discrete skin sites with independent control of vibration frequency, amplitude, and phase, while providing accurate control of stimulus's timing and location.

**Figure 1:**
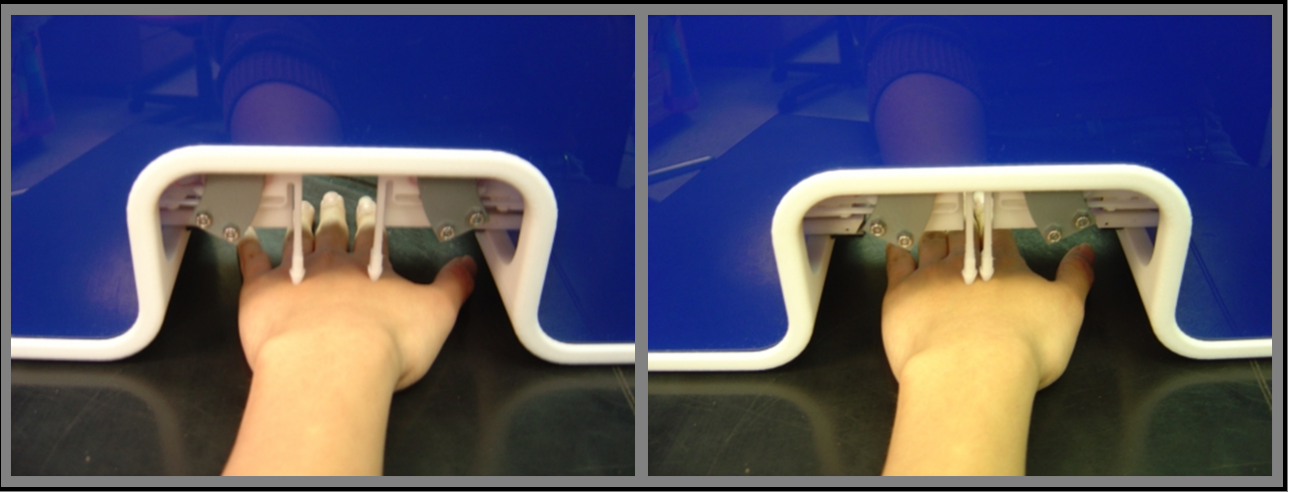
**Stimulus position on the dorsal surface of the left hand**. Probe tips detect the surface of the skin automatically. The stimuli were initially spaced 30 mm apart (left panel of figure) and the inter-stimulus distance was modified on a trial-by-trial basis based on the subject's performance. The minimal inter-stimulus distance possible was 5 mm with 5 mm diameter probe tips (right panel of figure).

During the experiment, the subject was seated in a chair with his/her left forearm on the table positioned comfortably to allow unimpeded access of the stimulator to the center of the dorsal surface of left hand (Figure 1). To ensure a stable hand position for the duration of the experiment, the subject was instructed to place their palm on the table surface as flat as possible, and a bead bag was applied to immobilize the wrist. The reasons that we selected the hand dorsum to receive the stimulation are: 1) the innervation density across this skin region remains relatively constant; 2) the surface is easily accessible and permits convenient stimulator placement; 3) use-dependent plasticity is minimized (i.e., the hand dorsum is, for the most part, used the same amount in daily activity by all subjects); and 4) it permits positioning of the subject's arm and hand in a comfortable and stable position for the full duration of an experimental session.

**Figure 2:**
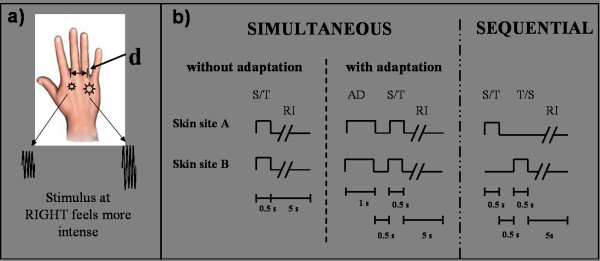
**Timing diagram of the protocol**. a) Two 25 Hz flutter stimuli, identical except for a constant difference in amplitude (standard stimulus (S): 100 μm vs. test stimulus (T): 140 μm peak-to-peak amplitude) were delivered. The stimuli were initially spaced 30 mm apart, and the inter-stimulus distance (d) was modified on a trial-by-trial basis based on subject performance. b) The task was performed under three conditions: 1) Simultaneous without adaptation: in each trial, the standard (S) and test (T) stimuli were delivered at the same time for 0.5 s. A 5 s delay including the subject response interval (RI) was imposed before onset of the next trial; 2) Simultaneous with dual-site adaptation: a pair of adapting stimuli (AD) (identical to the standard stimulus) was delivered first for 1 s at the same pair of sites as the test and standard stimuli. After a 0.5 s inter-stimulus interval, the test and standard stimuli were presented simultaneously; 3) Sequential: the standard and test stimuli were presented sequentially with a 0.5 s inter-stimulus interval. The order and loci of standard and test stimuli were randomized on a trial-by-trial basis.

A two alternative forced-choice (2AFC) tracking procedure was used to assess a subject's ability to discriminate between the amplitudes of two simultaneously delivered stimuli positioned at near-adjacent skin sites. Each run consisted of 20 trials. At the start of each trial, the two probe tips, 5 mm in diameter, were driven to the skin surface together and automatically stopped after skin detection. The tips were indented 500 um further to ensure good contact with the skin. The stimulus position and timing diagram of the protocols are shown in Figure 2. Two 25 Hz flutter stimuli, identical except for a constant difference in amplitude (standard stimulus: 100 μm vs. test stimulus: 140 μm peak-to-peak amplitude), were delivered (see Figure 2a). After each trial, the subject was queried as to which skin site received the more intense stimulus. Subjects were instructed to indicate their selection with a switch box with their free hand.

The stimuli were initially spaced 30 mm apart (see Figure 1; well above two-point discrimination limen on the hand dorsum; [6-8]), and the inter-stimulus distance was modified on a trial-by-trial basis based on the subject's performance. During the first 10 trials, a 1 up/1 down tracking paradigm was used, allowing a single correct answer to cause a 10% reduction in inter-stimulus distance in the subsequent trial. After one inaccurate response, the probe tips were moved 10% further apart. In the last 10 trials, a 2 up/1 down tracking algorithm was used in which two correct responses were required to decrease the inter-stimulus distance by 10%. The combination of two tracking algorithms in this manner allows the threshold to be determined much faster without compromising the results [8,14].

The task was performed under three conditions (see Figure 2b): 1) *Simultaneous stimulation without adaptation*: in each trial, the standard (S) and test (T) stimuli were delivered at the same time for 0.5 s. A 5 s delay including the subject response interval (RI) was imposed before onset of the next trial; 2) *Simultaneous stimulation with dual-site adaptation*: a pair of adapting stimuli (identical to the standard stimulus) was delivered first for 1 s at the same pair of sites as the test and standard stimuli. After a 0.5 s inter-stimulus interval, the test and standard stimuli were presented simultaneously; 3) *Sequential Stimulation*: the standard and test stimuli were presented sequentially with a 0.5 s inter-stimulus interval. The order and loci of standard and test stimuli were randomized on a trial-by-trial basis. The three run conditions were randomized on a subject-by-subject basis.

Repeated measures analysis of variance (ANOVA) was used to evaluate the difference of the subject's performance under three conditions. Data are presented as means and standard errors (SE). A probability of less than 0.05 was considered statistically significant.

## Results

**Figure 3:**
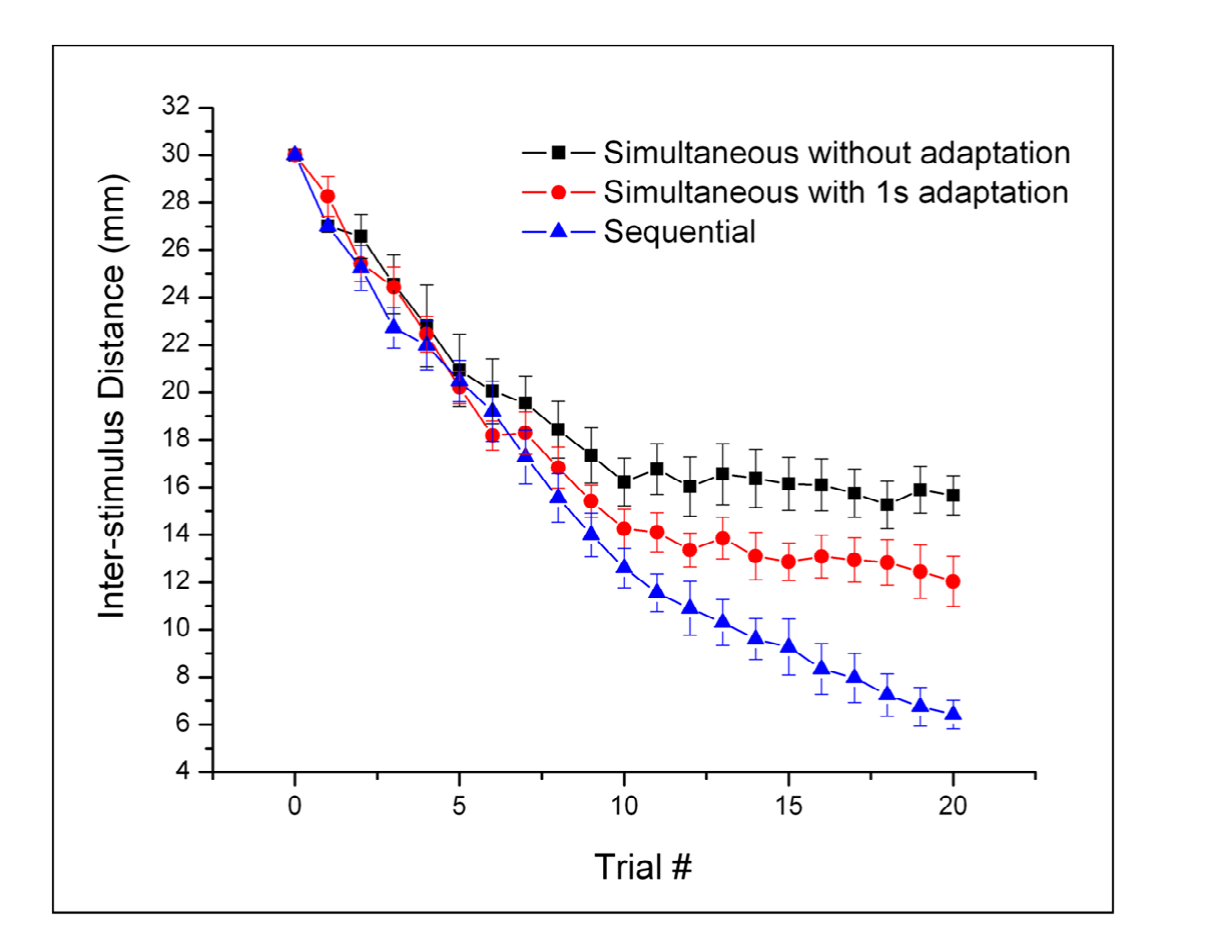
**The averaged response of tracking performance under three conditions**. The subjects' performance was degraded as the stimuli were moved closer together in the simultaneous condition but not in the sequential delivery of stimulation. Under the third condition – the simultaneous stimulus condition with adaptation – pre-exposure to a pair of flutter stimuli (adaptation) at the same locations as the standard and test stimuli improve a subjects' discriminative capacity.

A subject's ability to discriminate the intensity difference between two vibrotactile stimuli of fixed amplitudes at varying distances between stimulus sites was tracked to approach the inter-probe distance limit at which subjects could not reliably discriminate between the two stimuli. Figure 3 is a plot of the averaged response of tracking performance under three different conditions of stimulation. Each condition resulted in a significant change in tracking performance. Comparison of the data obtained in the sequential stimulation condition and the simultaneous stimulation condition demonstrates that the subjects' performance was degraded as the stimuli were moved closer together in the simultaneous condition, but not in the sequential delivery of stimuli. Note that when the inter-stimulus distance was decreased to approximately 16 mm (near the two-point limen for 25 Hz vibrotactile stimuli on the hand dorsum; [6-8]), discrimination performance became much worse. In contrast, for the sequential condition, subjects were able to correctly discriminate at all inter-stimulus distances, until the separation became 5 mm (minimal inter-stimulus distance possible with 5 mm diameter probe tips). Additionally, subjects' performance under the third condition – the simultaneous stimulation condition with adaptation – shows that pre-exposure to a pair of flutter stimuli (adaptation) at the same locations as the standard and test stimuli improve a subject's discriminative capacity. The data demonstrates a certain degree of consistency across subjects, as variability in the averaged plots of Figure 3 is relatively low (note error bars in plots).

**Figure 4:**
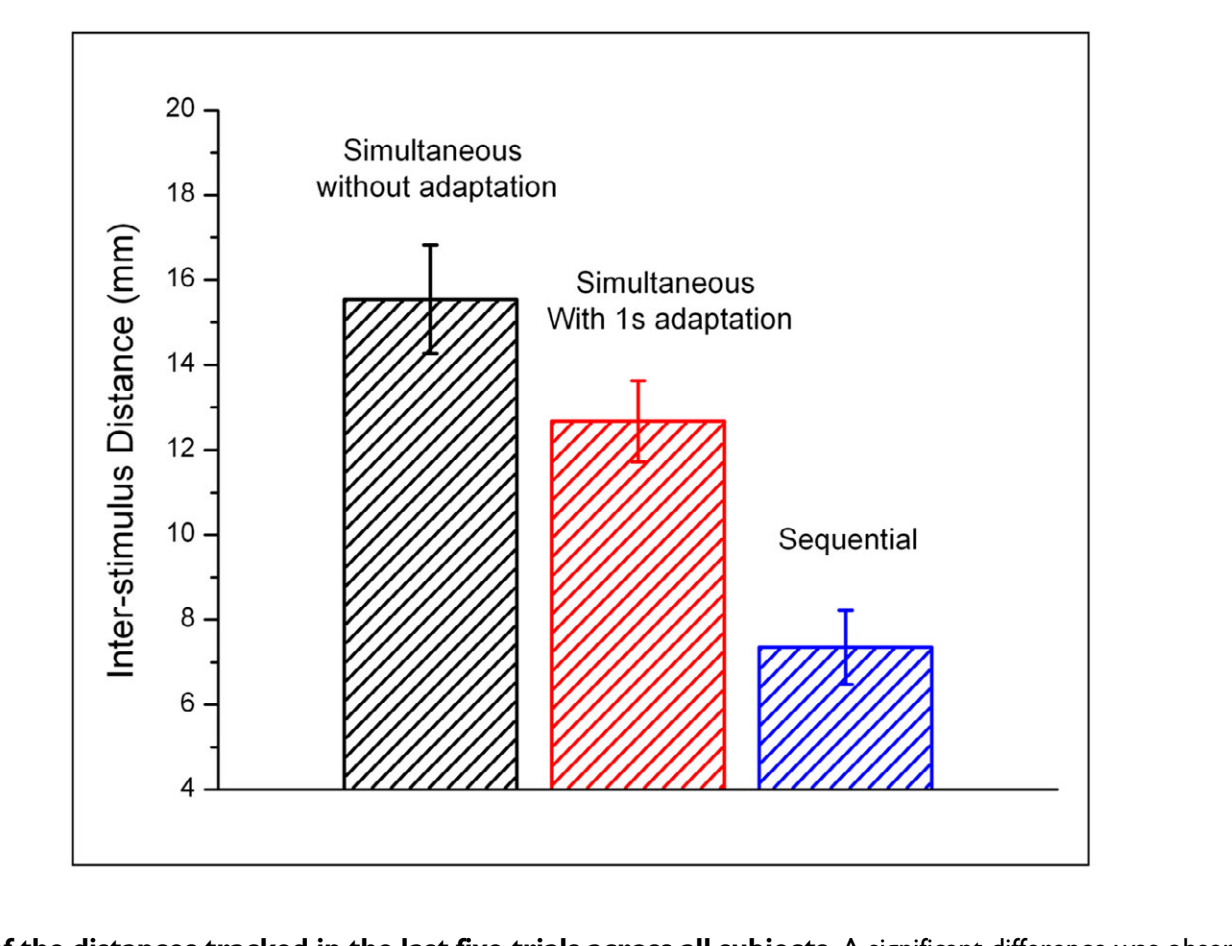
**Average of the distances tracked in the last five trials across all subjects**. A significant difference was observed in performance between the simultaneous stimulation without adaptation and the sequential conditions (p < 0.001). Adaptation resulted in a significant improvement (~20%) on simultaneous amplitude discrimination at small inter-stimulus distances (p = 0.034).

In order to more directly compare the responses measured under each of the stimulation conditions, the tracking values obtained from the last five trials across all subjects were averaged (Figure 4). A significant difference was observed in performance between the simultaneous without adaptation and sequential conditions (p < 0.001). Additionally, when compared to the simultaneous non-adapting condition, subjects' performance in the simultaneous discrimination task with adaptation was significantly improved by ~20% (p = 0.034).

## Discussion

In the present study, we investigated the effects of spatial acuity on amplitude discrimination between two flutter stimuli (25 Hz) delivered to the dorsal surface of the hand. The results show that subjects were able to discriminate the amplitude difference between two sequentially delivered stimuli at all inter-stimulus distances from 30 mm to 5 mm (the diameter of the probe tip). When stimuli were presented simultaneously, however, the subjects' ability to discriminate the same amplitude difference was significantly impaired as the inter-stimulus distance was reduced to 16 mm (near the two-point limen). These results are consistent with a previously published report that demonstrated that amplitude discrimination capacity was significantly worse when inter-stimulus distances were reduced from 30 mm to 5 mm [14]. In a task that tracked only a subject's ability to discriminate amplitude differences, Tannan et al found a significant difference in amplitude discrimination capability when the stimuli were delivered simultaneously vs. sequentially at near adjacent skin sites (10 mm or less). Additionally, the results were consistent with the two-point discriminative capacity previously reported for the hand dorsum (16 mm, 17 mm, 20 mm, and 12 mm respectively for four subjects) by Tannan et al [6]. However, in that study, the inter-subject variability was reported to be much higher (20% vs. 10% of the threshold value), and we suspect that the increased variability in that task was due to the subjective nature of the task. In other words, variability for the findings in this report were lower principally due to the increased objectivity of an amplitude discrimination task that fails due to a decreased spatial discriminative capacity rather than delivering two points to the skin and challenging the subject to only determine whether they felt one or two points.

Sequential and simultaneous test conditions were delivered in order to directly assess the impact that inter-stimulus distance had on a subject's amplitude discrimination capacity. The comparison between sequential and simultaneous stimulus conditions demonstrated that the degradation of amplitude discrimination capacity in the simultaneous stimulus condition was possibly solely due to the subject's inability to discriminate between two points when they were located in near proximity. LaMotte and Mountcastle [16,17] stated that the ability of a subject to accurately localize a flutter stimulus on the skin is determined by the locus and clarity of the neuronal population response within the topographically organized SI network. When two stimuli are positioned close together on the skin, the activity in the two neuron populations evoked by the two stimuli in the cortex may tend to overlap. As a result, subjects may perceive only one, instead of two distinct sensations. If this is the case, the distance between two stimuli tracked in the simultaneous stimulus condition may be equivalent to the spatial metric that traditional TPD tests were intended to measure.

An important distinction between the protocol used in this study and the traditional two-point discrimination tasks is that the amplitudes of the two stimuli were significantly different, and it is important to consider the spatial extent that larger amplitude stimuli may (or may not) occupy. Simons et al [18,19] imaged the optical intrinsic signal of the SI responses evoked by vibrotactile stimulation with different amplitudes in non-human primates. They found that as the stimulus amplitude was increased, the activity within the activated region of SI cortex progressively increased although the spatial extent of the activated region remained relatively constant. Rather, with increasing stimulus amplitude and duration, the region surrounding the activated cortical field became less active (or more inhibited), suggesting that more intense and longer duration stimuli would result in more spatially resolved stimuli. Results of the present study appear to be consistent with the findings of Simons and colleagues such that all subjects demonstrated improved discrimination in the simultaneous stimulus condition when the stimulus sites were pre-exposed to 1 s adapting stimulation.

The effects of an adapting stimulus on the perception of subsequent stimuli – particularly the reduction in sensation – have been characterized in some detail [20-25]. However, only a relatively small number of studies have assessed the impact that prior exposure to vibrotactile stimuli has on spatial localization or the spatial acuity necessary to discriminate between two points on the skin, and all of these studies demonstrated that adaptation improved spatial acuity [7,8,26,27]. This improvement was originally proposed to be due to the improved spatial clarity between topographically distinct regions of SI cortical activity [16,17]. Two recent reports have examined the effects of stimulus duration-dependent changes on a subject's ability to spatially localize a stimulus. Tannan et al [8] demonstrated that the performance of neurologically healthy human adults on a spatial localization task undergoes a prominent change with pre-task exposure to an adapting stimulus. In that study, it was determined that adaptation with a longer duration (5 s) vibrotactile stimulus resulted in an approximately 2-fold improvement in spatial localization performance over that achieved with a shorter (0.5 s) stimulus. It was proposed that this observed improvement in spatial localization was due to the enhanced spatial funnelling of the population-level response of contralateral primary somatosensory cortex (SI) – a robust phenomenon that is at least in part due to GABAergic inhibitory neurotransmission [28] and has been demonstrated using comparable stimulus conditions in neuroimaging studies of anesthetized non-human primates [18,19,29]. A subsequent report strengthened this argument by demonstrating that neurologically compromised subjects with a known GABAergic deficiency (adults with autism) showed no such improvement at the same spatial localization task with adaptation [30]. Thus, there seems to be some evidence that spatial acuity does improve in a stimulus-dependent and GABA-mediated manner that undoubtedly impacts the spatial contrast of cortical activity evoked by vibrotactile stimuli. Changes in the responsivity of neurons have been proposed to underlie the cortical mechanisms for stimulus feature extraction and may be important in the improvements observed in spatial discrimination such as those described above (for review see [31]). This enhancement of discrimination capacity could be due, at least in part, to the moment-to-moment changes that occur in the spatio-temporal patterns of response with repetitive vibrotactile stimulation.

We speculate that the observed improvement of subjects' performance in this study with adaptation is solely due to the effects of adaptation on spatial acuity. It is important to note that in this study, instead of tracking an amplitude difference (as in more commonly performed amplitude discrimination tasks), a constant amplitude difference, which is well above normal subject's amplitude discrimination threshold [14], was maintained while the inter-probe distance was tracked. The subjects' excellent performance under the stimulus condition in which stimuli were delivered sequentially suggests that discriminative capacity (in the simultaneous stimulation condition) was predominantly impacted by the spatial parameters imposed by the inter-stimulus distance. As a result, when two stimuli were delivered simultaneously and in near-proximity, the effects of pre-exposure to dual-site adapting stimuli would be to facilitate the discriminative aspect affected by spatial acuity, but not necessarily facilitate what would normally be an easy amplitude discriminative task. Thus, any adaptive effects on the amplitude discriminative task – which have been reported in several studies [15,32-34] – could most likely be regarded as having little impact on the results in this study.

## Conclusion

Subjects were not able to discriminate between two amplitudes of vibrotactile stimulation simultaneously delivered to the skin as the inter-stimulus distance approached the limit of a subject's spatial acuity. The inter-stimulus distance metric obtained from the study is robust across the subjects studied thus far (i.e., low variance between individual performance) and can be obtained relatively quickly (about three minutes). The strongest candidate responsible for the improvement in performance observed with adapting stimulation appears to be GABAergic mediated lateral interactions. Performance on the task, as well as improvement on the task with adaptation would most likely be impaired if GABAergic function in a subject (or subject population) were systemically altered, and thus, the methods described could provide an effective set of measures for assessing systemic cortical alterations in such subject populations.

## Abbreviations

TPD: two point discrimination; 2AFC: two alternative forced-choice; TPS: two-point stimulator; ANOVA: analysis of variance.

## Competing interests

The author(s) declare that they have no competing interests.

## Authors' contributions

ZZ participated in the design of the study, carried out the data collection and analysis, and drafted the manuscript. VT participated in the design of the study and revision of the manuscript. JH assisted in design of the protocol and in the data collection. RD contributed to the design of the experiment. MT played a role in the design of the study, the coordination of the study and drafting the manuscript. All authors read and approved the final manuscript.
